# Influence of *Leishmania (Viannia) braziliensis* infection on the attractiveness of BALB/c mice to *Nyssomyia neivai* (Diptera: Psychodidae)

**DOI:** 10.1371/journal.pone.0214574

**Published:** 2019-04-01

**Authors:** Flávia Benini da Rocha Silva, Danilo Ciccone Miguel, Vicente Estevam Machado, Wanderson Henrique Cruz Oliveira, Thais Marchi Goulart, Christiann Davis Tosta, Hildete Prisco Pinheiro, Mara Cristina Pinto

**Affiliations:** 1 Departamento de Ciências Biológicas, Universidade Estadual Paulista (UNESP), Faculdade de Ciências Farmacêuticas, Câmpus Araraquara, Araraquara, São Paulo, Brasil; 2 Departamento de Biologia Animal, Universidade Estadual de Campinas (UNICAMP), Campinas, São Paulo, Brasil; 3 Instituto Federal de Educação, Ciência e Tecnologia de São Paulo (IFSP), Campus Matão, Matão, São Paulo, Brasil; 4 Departamento de Estatística, Universidade Estadual de Campinas (UNICAMP), Campinas, São Paulo, Brasil; Instituto Oswaldo Cruz, BRAZIL

## Abstract

**Background:**

Phlebotomine sand flies are vectors for several pathogens, with *Leishmania* being the most important. In Brazil, the main aetiological agent of American cutaneous leishmaniasis (ACL) is *Leishmania (Viannia) braziliensis*, and *Nyssomyia neivai* is one of its main vectors in São Paulo state and other areas of South America. Similar to other haematophagous insects, sand flies use volatile compounds called kairomones to locate their hosts for blood meals. A possible increase in the attractiveness of hosts infected with *Leishmania infantum* to their vectors has been demonstrated. In the present study, we aimed to investigate whether *L*. *braziliensis*-infected hosts present higher attractiveness to *Ny*. *neivai* and to identify differences in the volatile compounds released by infected and uninfected mice.

**Results:**

Behavioural experiments in which sand fly females directly fed on infected or uninfected mice showed no significant differences in the attractiveness of the mice or the blood volume ingested. Y-tube olfactometer bioassays also revealed no significant differences in the attractiveness of these hosts to *Ny*. *neivai*. No differences were observed in the profiles of the volatile compounds released by the two groups of mice. However, PCA and cluster analysis were able to classify the 31 identified compounds into three clusters according to their abundances. This classification showed a possible role for individual variation in the absence of differences in volatile profiles and attractiveness between infected and uninfected mice.

**Conclusion:**

In this first cross-sectional study with an aetiological agent of ACL, there were no statistically significant differences in the attractiveness of infected hosts to their vector.

## Introduction

Leishmaniasis is a zoonosis caused by flagellated protozoa of the genus *Leishmania*, which are transmitted by haematophagous insects called phlebotomine sand flies. Leishmaniasis cases can be clinically divided into visceral and cutaneous leishmaniasis, which are considered to be neglected tropical diseases; these forms of leishmaniasis are endemic in 97 countries worldwide, and one billion people live in areas with a risk of transmission [1]. In Brazil, the main aetiological agent of American cutaneous leishmaniasis (ACL) is *Leishmania (Viannia) braziliensis*, and autochthonous cases have been confirmed all over the country [2]. Among sand fly species, *Nyssomyia neivai* is one of the main vectors of the aetiological agent of ACL in the state of São Paulo and has been implicated as a vector of ACL in other areas of South America [3,4]. In addition to reports of natural *L*. *braziliensis* infection in *Ny*. *neivai*, a study evaluating parameters of vector capacity, such as the duration of the gonotrophic cycle, the proportion of females that fed on hamsters, the rate of infection by *L*. *braziliensis* and the duration of the extrinsic incubation period, reinforced the vectorial role of *Ny*. *neivai* [5].

During their blood meal, the salivary content of sand flies can cause a strong inflammatory reaction, exacerbating *Leishmania* infection [6,7]. The blood volume ingested by sand flies may determine important factors for insect survival, such as the number of eggs, the frequency of host searching, and the number of bites, in addition to influencing the quantity of pathogens acquired by the insects from an infected host [8].

Haematophagous insects use volatile compounds released by their hosts, called kairomones, to locate sources of blood meals. Volatile compounds that are known to be released by vertebrate hosts can be used as baits and applied to monitor and/or control insects involved in disease transmission [9]. With respect to sand flies, field and laboratory studies involving kairomones have focused mainly on CO_2_, lactic acid and 1-octen-3-ol [10–14].

Recent studies involving hosts infected with *Plasmodium* spp. have shown increased attractiveness to malaria vectors and differences in the volatile compounds released by these hosts [15–18]. The same pattern has been suggested for the aetiological agent of visceral leishmaniasis, since increased attractiveness of *Leishmania infantum-*infected hosts to the parasite’s vector *Lutzomyia longipalpis* has been demonstrated [19,20]. In a cross-sectional study, when different uninfected and infected animals were evaluated in the same moment, it was suggested that differences in the attractiveness of *L*. *infantum-*infected hosts to *L*. *longipalpis* are due to the different volatile compounds released by these hosts [19]. However, in a longitudinal study, when the same animal was evaluated before and after the infection, individual variations were shown to be very important, and not all animals infected with *L*. *infantum* exhibited increased attractiveness to *L*. *longipalpis* [20]. Until now, among more than 30 species of *Leishmania*, no study on this topic has addressed species that cause cutaneous leishmaniasis.

Although there is evidence that *Leishmania* infection may influence the quantity of blood ingested and the frequency of sand fly blood meals, thereby increasing the transmission rate of the parasite, it is not known whether parasites can affect host attractiveness to sand flies [21,22].

In extracts of volatile compounds collected by solid-phase microextraction (SPME) from hairs of infected and uninfected dogs, six compounds were identified that may be considered to be potential biomarkers of infection by *L*. *infantum chagasi* [23].

Although the SPME technique was initially developed for the analysis of atmospheric air pollutants, its use has expanded to other samples [24]. Extraction by SPME presents the advantages of not involving solvents, reducing the time needed for sample preparation, and yielding a higher sensitivity, as this approach is mainly associated with gas chromatography-linked mass spectrometry (GC-MS) for the separation and identification of analytes [25].

Increased attractiveness of infected hosts could favour the transmission cycle of the parasites, and the identification of different volatile compounds profiles may reveal potential biomarkers of infection or lead to the development or enhancement of baits for the monitoring/control of vectors, especially in endemic regions [26].

This is the first study focusing on one of the most relevant aetiological agents of cutaneous leishmaniasis in the Americas, i.e. *Leishmania braziliensis*. We aimed to investigate whether *Ny*. *neivai* is differentially attracted to BALB/C mice infected with *L*. *braziliensis* and investigate released volatile compounds during insect-mammalian host interaction. Moreover, blood volumes ingested by *Ny*. *neivai* from both infected and uninfected mice were assessed.

## Materials and methods

### Sand flies

The insects were obtained from the colony maintained since 2013 in the Biological Sciences Department of the School of Pharmaceutical Sciences in Araraquara, São Paulo state, Brazil [27,28].

### *Leishmania* infection

*Leishmania braziliensis* (MHOM/BR/94/H3227), which was isolated from a cutaneous leishmaniasis patient, was kindly donated by Dr. Maria Jania Teixeira (Federal University of Ceará, Brazil) and maintained in the laboratory for experimental mouse infections in the Department of Animal Biology–Biology Institute, UNICAMP. To infect the animals, 10 μL of saline containing 10^5^ stationary-phase promastigotes was injected subcutaneously into the posterior paws of female BALB/c mice [29,30].

Isogenic mice were infected at 2–3 months old and used approximately two months after infection, when oedema could be observed at the site of infection. All animals were maintained under the same conditions, and during the trials, they were anaesthetized intramuscularly with ketamine 10% (120 mg kg^-1^) and xylazine 2% (5 mg kg^-1^) [31].

After the tests were carried out, the parasite was reisolated from the infection site, and promastigote forms of *L*. *braziliensis* were visualized in complete M199 culture medium (Sigma-Aldrich).

### Infected and uninfected mice exposed to sand fly bites and blood volume

**Fig 1:**
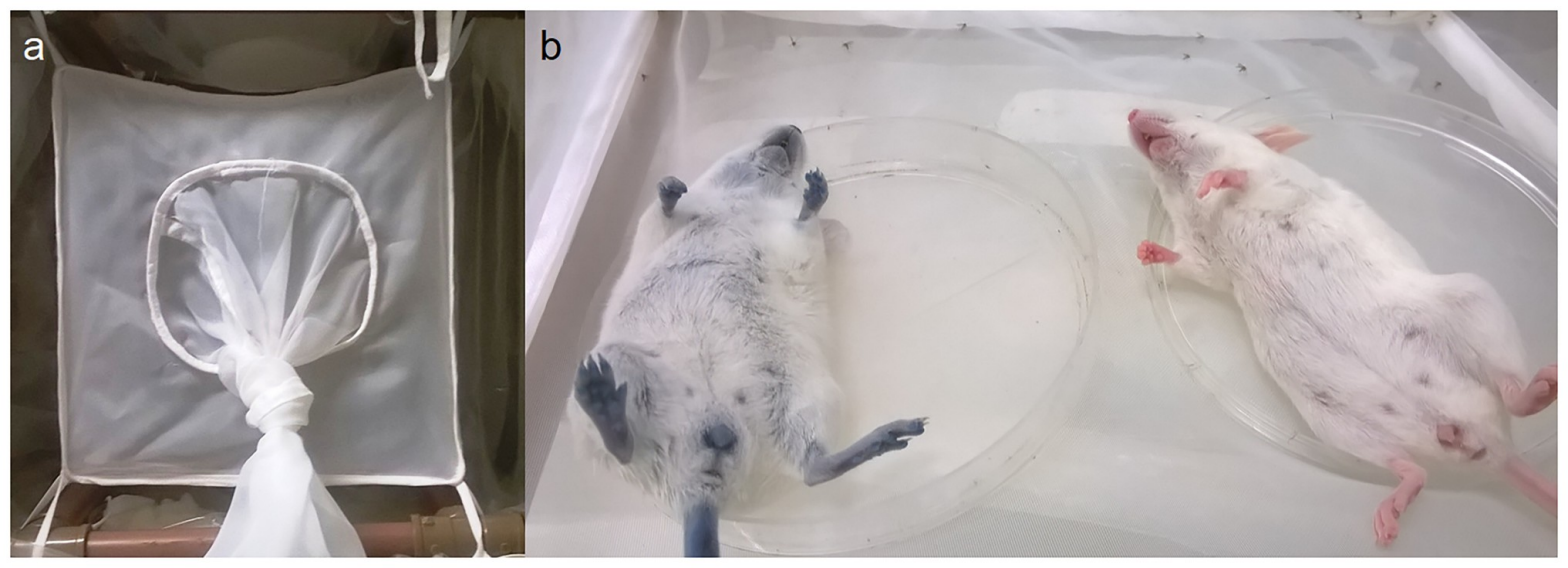
Bioassay in a Barraud cage to investigate the attractiveness of infected and uninfected mice to *Ny*. *neivai*. (a) Barraud cage; (b) mice in Petri dishes inside the cage—injected or not with Evans Blue dye.

To evaluate the attractiveness of infected and uninfected mice to *Ny*. *neivai*, two animals were anaesthetized and housed simultaneously for one hour inside a Barraud cage (30 × 30 × 30 cm) (Fig 1a) with 30 male and 30 female sand flies (5–7 days post emergence) without a previous blood meal. The sand flies had access to a 30% sucrose solution fed *ad libitum* until 6 hours before the experiment. Attractiveness was measured indirectly by the number of females that fed on each mouse.

To distinguish the blood source of the fed females, one mouse was previously injected with Evans Blue dye (EB) diluted in PBS (200 mg kg^-1^), and the other mouse was injected only with PBS (Fig 1b) [32]. The injection of EB dye did not influence the attractiveness of the mice to the insects, as demonstrated for *Aedes aegypti* [32]. In order to confirm that the EB dye had no effect on attractiveness to the sand flies, the experiments were replicated 16 times, with 8 replicates for each condition: infected mice+EB dye and uninfected mice+EB dye. Different insects and different mice were used in each replicate. In addition, a logistic regression analysis was carried out considering as response the probability of a sand fly to bite the infected mouse among those sand flies which have bitten any mice, with the dye effect as covariate in the model [33,34].

To estimate the blood volume ingested by each sand fly, we adapted the methodology established for *Aedes aegypti* [32] due to the smaller size of sand flies.

First, one mouse was injected with EB diluted in PBS (200 mg kg^-1^), and another mouse was injected with the same volume of PBS. After 10 minutes, 10 μL of blood from each mouse was collected and diluted in 250 μL of deionized water. Half of this volume was serially diluted in deionized water (1:2), and 100 μL of each dilution was transferred to a 96-well plate to evaluate the absorbance at 540 and 620 nm, which are the wavelengths of maximum absorbance for haemoglobin and EB, respectively. Standard curves of absorbance *versus* blood volume were generated for the two wavelengths, and their respective linear equations were obtained.

After each attractiveness trial, the fed females were individualized and macerated in micro tubes containing 125 μL of deionized water. After homogenization, 100 μL from each micro tube was transferred to a 96-well plate for absorbance measurement at 540 and 620 nm. Individual absorbance values were applied to the linear equations to estimate the blood volume ingested by each sand fly female.

### Attractiveness of infected and uninfected mice in Y-tube olfactometer

**Fig 2:**
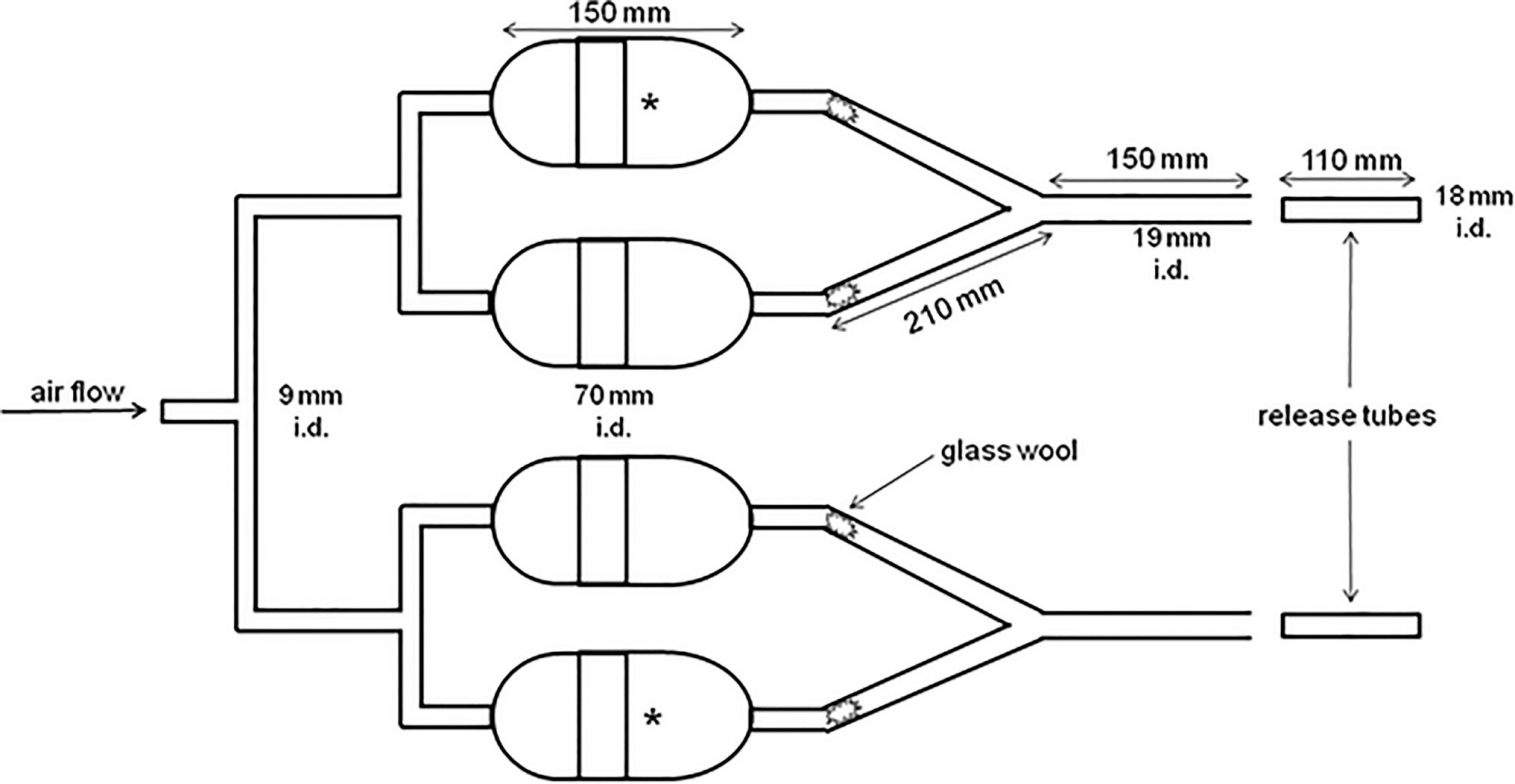
Illustration of the use of a Y-tube olfactometer to evaluate the attractiveness of infected and uninfected mice to *Ny*. *neivai*. * Initial position of the mouse. Arrows indicate release tubes in which female sand flies were placed.

Some behavioural tests were also performed in a Y-tube olfactometer to evaluate the attractiveness of the infected and uninfected mice to the sand flies. Tests were performed with two Y-tube olfactometers simultaneously: one with an infected mouse and the other with an uninfected mouse. The experiment was repeated three times with different animals. One arm of each Y-tube was left empty as a negative control. For every 10 sand fly females tested, the positions of the mouse and control arms of each olfactometer were inverted to avoid position bias (Fig 2).

Anaesthetized mice were placed in the Y-tube olfactometer, and an air flow (2.0 L minute^-1^) passed through the olfactometer to carry the odour stimulus to the sand flies. For each mouse, 30 sand fly females without a previous blood meal were individually placed in the release tubes and evaluated by the same researcher (FBRS) for a maximum observation period of 4 minutes each. In total, 90 females were used for the infected mice, and 90 females were used for the uninfected mice. The female sand flies were evaluated for activation (the number of sand flies leaving the release tube) and attraction (the number of sand flies reaching the arm of the odour source). All experiments began at 17:00 h.

### Volatile compounds

Volatile compounds released by the two groups of animals were extracted by the SPME technique and analysed by gas chromatography-linked mass spectrometry (GC-MS). Prior to analysis of volatile compounds, standardization was performed for fiber selection and the time of extraction, using a quantity of fur from the mice to collect the volatiles [23,35].

After standardization, we collected 100 mg of hair from the dorsal region of each mouse (infected and uninfected) with a razor blade. The fur was conditioned in a 20 mL headspace vial, and a fiber coated with polydimethylsiloxane/divinylbenzene (PDMS/DVB) was inserted into the vial. The PDMS/DVB fiber (Stableflex—Supelco, Bellefonte, PA, USA) was exposed to the sample for 30 minutes at 90 °C. An empty vial was used as a control, and extractions were carried out in triplicate for each group (infected and uninfected).

After the extractions, the fibers were inserted into the gas chromatograph (GC-MS QP2010 Plus—Shimadzu) for five minutes to desorb volatile compounds at 240°C in splitless mode. The volatile compounds in the samples were analysed in an RTx-5MS capillary column (30 m × 0.25 mm i.d.; 0.25 μm) with a carrier gas (He) flow rate of 1.0 mL minute^-1^. The temperature schedule was 70°C for 2 minutes, 2°C minute^-1^ until 120°C, 120°C for 20 minutes, 4°C minute^-1^ until 250°C, and 250°C for 5 minutes (total of 84 minutes). The temperatures of the ion source and transfer line were adjusted to 250°C, with an electron impact energy of 70 eV and spectrum scanning from 40 to 600 m/z.

Volatile compounds were identified by comparing mass spectra to the library of the machine (NIST 08, NIST 98v101, Wiley MS 229 and FFNSC 1.3) and by comparing the retention indexes to those of a mixture of C8-C20 n-alkanes.

### Data analysis

For the cage experiments, a binary logistic model was used to analyse the effects of the dye and *Leishmania* infection on the proportions of sand flies that fed on each group of mice. For the Y-tube experiments, the Mantel-Haenszel Chi Square test was used to analyse the probability that the sand flies would be attracted to the control or to uninfected or infected mice. The test was carried out as an aggregate of all three experiments and individually. In a second step, a logistic regression model was generated in SAS 9.3 (SAS Institute, Cary NC) with the LOGISTIC procedure using the probability of attractiveness of the mice and the control, considering the following covariates: infected or uninfected mice and experiment. The interaction between the covariates was also analysed. For volatile compounds analysis, the relative areas of the identified peaks were subjected to principal components analysis (PCA) and Ward’s cluster analysis (XL STAT software).

### Ethics statement

All animal experiments were conducted according to the Guidelines for Animal Experimentation of the Conselho Nacional de Controle de Experimentação Animal (CONCEA). This study was approved by the Ethics Committee on the Use of Animals (CEUA) of FCFAr/UNESP (Protocol 52/2015).

## Results

### Infected and uninfected mice exposed to sand fly bites and blood volume

When attractiveness was measured by direct blood feeding of females in Barraud cages, the number of fed females was low in all of the assays. Despite this low response of the sand fly females, a binomial exact test showed that there was no significant difference between the infected and uninfected groups in any of the replicates or in the aggregated results (Table 1).

**Table 1:** Number of females that fed on each mouse in the different replicates. I + EB dye: infected mice injected with Evans Blue dye; Un + PBS: uninfected mice injected with PBS; I + PBS: infected mice injected with PBS; Un + EB dye: uninfected mice injected with Evans Blue dye; nr: no response.

	**I + EB dye**	**Un + PBS**	**nr**	p-value*
**1**	5	1	24	0.22
**2**	5	2	23	0.45
**3**	4	2	24	0.68
**4**	3	4	23	1.00
**5**	3	3	24	1.00
**6**	0	1	29	1.00
**7**	6	7	17	1.00
**8**	10	4	16	0.18
**Subtotal**	**36**	**24**	**180**	**0.15**
	**I + PBS**	**Un + EB dye**	**nr**	**p-value**
**1**	2	0	28	0.50
**2**	0	2	28	0.50
**3**	5	2	23	0.45
**4**	1	1	28	1.00
**5**	1	5	24	0.22
**6**	2	2	26	1.00
**7**	5	4	21	1.00
**8**	7	9	14	0.80
**Subtotal**	**23**	**25**	**192**	**0.88**
**Total**	**59**	**49**	**372**	**0.38**

* p-value for the exact test of H_0_:p = 0.5 *vs* H_1_:p≠0.5 where p = the probability of a sand fly to bite the infected mouse among those sand flies which have bitten any mice.

Moreover, by adjusting a binary logistic model with the aggregated results, we confirmed that there is no effect of EB dye (Wald chi-square = 1.55, df = 1, p = 0.21) or infection with *L*. *braziliensis* (Χ^2^ = 0.94, df = 1, p = 0.33) in the attractiveness.

**Fig 3:**
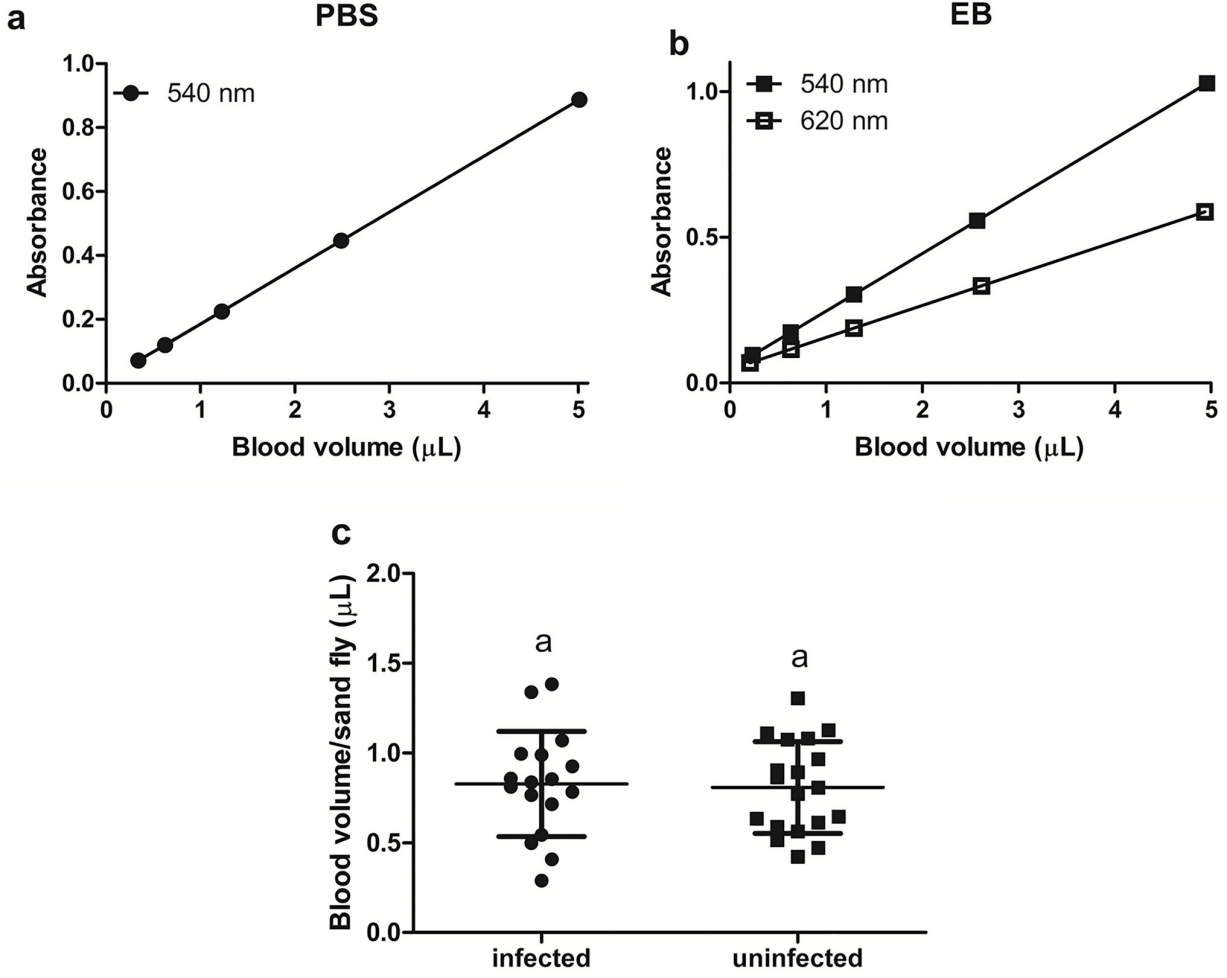
Standard curves of absorbance *versus* blood volume. (a) blood with PBS and (b) blood with EB; (c) blood volume ingested per sand fly from infected and uninfected mice. Means presented with the same letters are not significantly different (p>0.05).

After the attractiveness trials in Barraud cages, the blood volume ingested by each female sand fly was estimated according to previously obtained standard curves (Fig 3a and 3b).

From these standard curves, we observed no difference in blood volume, with means of 0.83 (±0.07) μL and 0.81 (±0.06) μL of blood ingested by the females from infected and uninfected mice, respectively (t = 0.21; df = 32; p = 0.83) (Fig 3c).

### Attractiveness of infected and uninfected mice in Y-tube olfactometer

Attractiveness assays using Y-tube olfactometers revealed a higher number of insects that responded to any mouse (61/121; 50%) when compared with the total number of blood fed females in the Barraud cage experiments (108/480; 22,5%).

**Fig 4:**
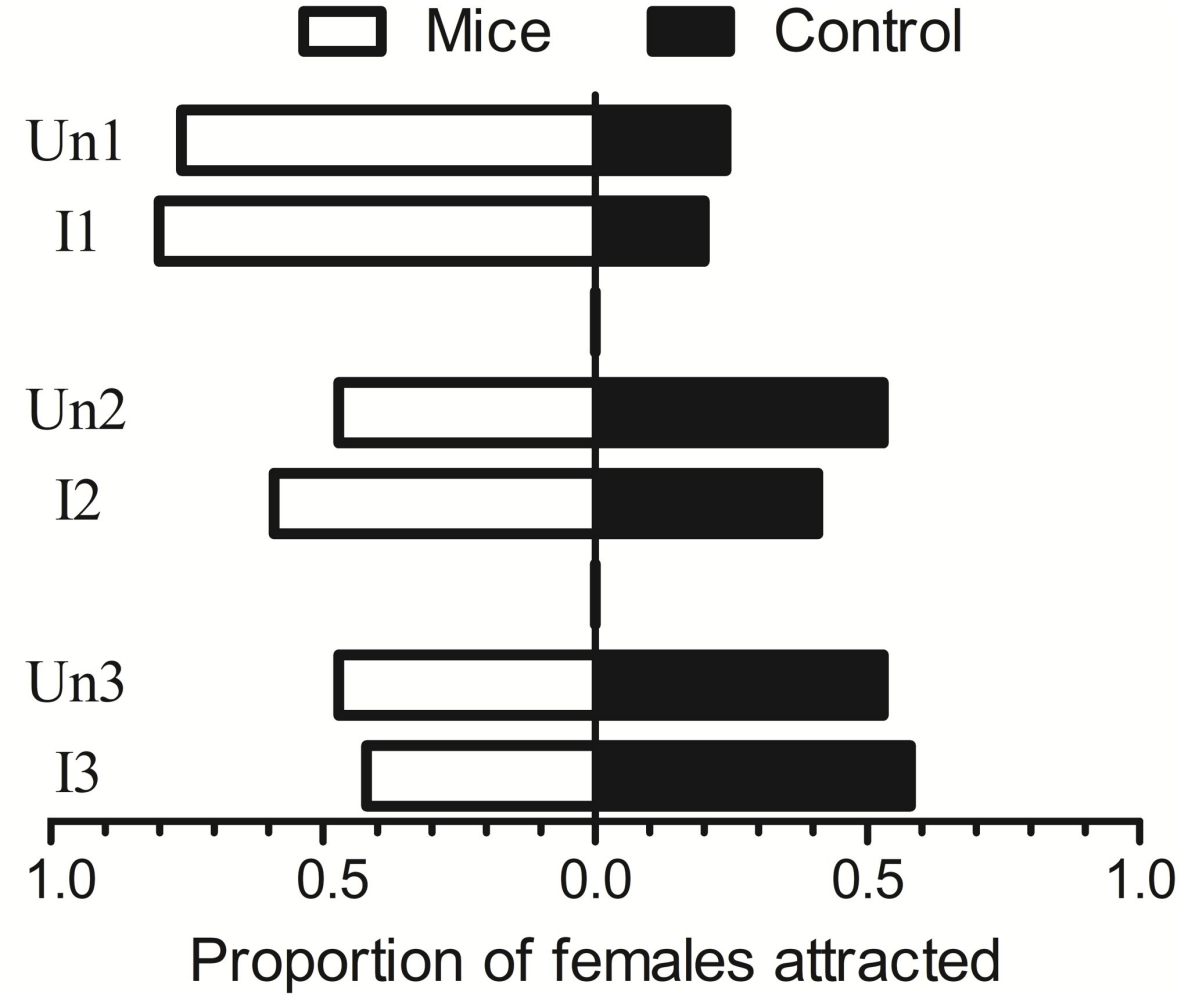
Proportions of females attracted to uninfected (Un1, Un2, and Un3) and infected (I1, I2, and I3) mice in Y-tube olfactometer trials; n = 30/mouse.

The results of the Mantel-Haenszel Chi Square test for the Y-tube olfactometer results showed no significant differences between the number of insects attracted to the control and the number of insects attracted to the mice (infected or uninfected), controlling for experiment (Χ^2^ = 0.14, df = 1, p-value = 0.70; n = 103). The results for each experiment were as follows: Experiment 1: Χ^2^ = 0.06, df = 1, p-value = 0.80, n = 37; Experiment 2: Χ^2^ = 0.46, df = 1, p-value = 0.50, n = 32; Experiment 3: Χ^2^ = 0.07, df = 1, p = 0.79, n = 34 (Fig 4).

Moreover, after adjusting the data from the Y-tube experiments to a logistic regression model with interaction between the covariates (infected or uninfected mice and experiment), we observed no interaction effect between the covariates (Χ^2^ = 0.46, df = 2, p-value = 0.79). In the next step, the model was performed without the interaction between the covariates and no significant difference between the sand flies attracted to mice or to control was observed (Χ^2^ = 0.15, df = 1, p-value = 0.70); however, there was a significant difference between experiments (Χ^2^ = 8.80, df = 2, p-value = 0.01).

### Volatile compounds

After prior standardization of the type of fiber coat and the time of extraction, we extracted volatile compounds from samples of fur obtained from infected and uninfected mice.

**Fig 5:**
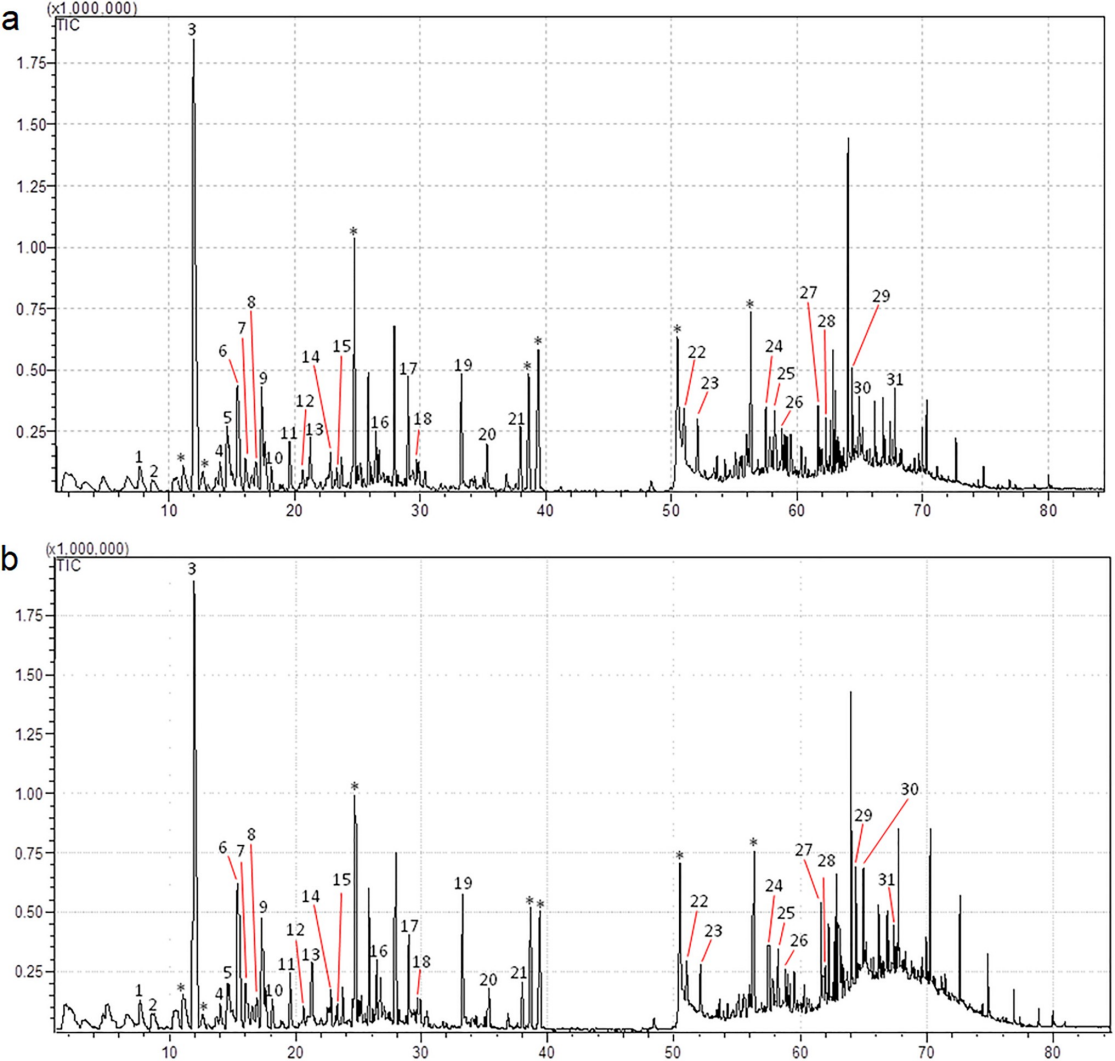
Chromatogram of volatile compounds profiles obtained from analysis of fur samples from uninfected (a) and infected (b) mice. The identified volatile compounds are numbered, and * indicates external contaminants.

From the obtained chromatograms, we observed similar peak profiles for volatile compounds from uninfected and infected mice, with variation in only the intensity and area of the peaks (Fig 5).

Analysis of mass spectra and posterior comparison of retention index values enabled the identification of 31 compounds extracted from both infected and uninfected mice (Table 2).

**Table 2:** Volatile compounds tentatively identified based on retention time (in ascending order) and relative areas for infected and uninfected mice.

Peak no.	Retention time (min)	Compound	Area (%) infected	Area (%) uninfected
**1**	7.67	octanal (a, b, c, d, e)	1.87	2.22
**2**	8.69	limonene (d)	0.99	1.07
**3**	11.95	nonanal (a, b, c, d, e)	36.25	36.37
**4**	14.05	*cis*-verbenol (e)	1.68	3.77
**5**	14.77	2-nonenal (c, d, e)	0.83	1.36
**6**	15.43	1-nonanol (e)	8.04	7.32
**7**	16.06	naphthalene (c)	2.93	3.05
**8**	16.95	(-)-myrtenol	3.28	4.87
**9**	17.36	decanal (a, b, d, e)	5.10	4.81
**10**	17.61	verbenone (e)	2.16	3.93
**11**	19.59	carvone	2.64	3.13
**12**	20.61	(E)-2-decenal (e)	0.46	0.58
**13**	21.29	nonanoic acid (e)	1.77	0.92
**14**	22.84	tridecane (b, c)	1.18	1.07
**15**	23.34	undecanal (c, d, e)	0.75	0.60
**16**	26.72	*cis*-8-undecen-1-al	0.91	0.91
**17**	29.01	tetradecane (b, c, e)	7.21	7.27
**18**	29.71	dodecanal (c, e)	0.61	0.52
**19**	33.26	geranylacetone (d, e)	5.23	3.30
**20**	35.29	dodecanol (c, e)	1.43	1.21
**21**	37.96	pentadecane (b, c, d, e)	2.23	2.07
**22**	51.00	hexadecane (b, c, d, e)	1.87	1.64
**23**	52.07	tetradecanal (e)	1.21	1.00
**24**	57.50	heptadecane (b, c, e)	1.62	1.31
**25**	58.20	pentadecanal (e)	0.89	0.77
**26**	58.80	6-phenyldodecane	0.70	0.57
**27**	61.67	octadecane (b, c, e)	1.37	0.89
**28**	62.28	hexadecanal	0.84	0.55
**29**	64.38	hexadecanol (e)	1.59	1.13
**30**	64.96	nonadecane (b, c, e)	1.16	0.88
**31**	67.78	eicosane (b, e)	1.20	0.92

a, b, c, d, e: volatile compounds previously found in mice (a: Röck et al., 2006), dogs (b: Oliveira et al., 2008; c: Magalhães-Junior et al., 2014), and humans (d: Dormont et al., 2013; e: Tavares, 2016).

Among the 31 identified compounds, 11 presented a higher content in uninfected mice since they demonstrated a larger relative area in this group of mice. After excluding contaminants, the relative areas of the identified compounds were subjected to PCA to verify similarities and differences between volatile compounds from the different groups of mice and to cluster analysis to classify the compounds.

**Fig 6:**
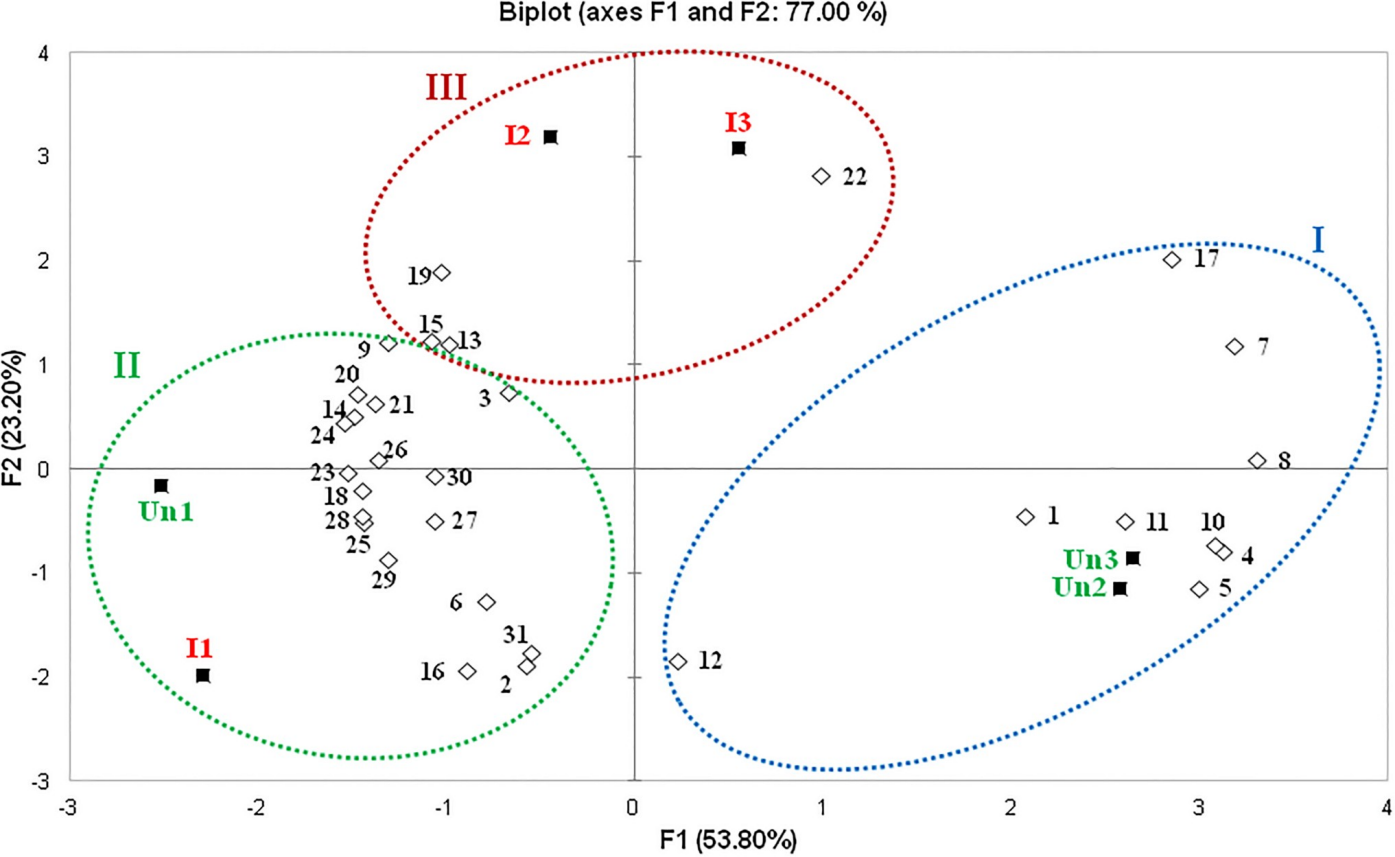
Principal component analysis and classification of clusters (I, II and III) of identified volatile compounds extracted from the different uninfected (Un1, Un2 and Un3) and infected (I1, I2 and I3) mice.

PCA of the first two principal components (F1 and F2) explained 77% of the total variability observed in the volatile compounds identified for the groups of infected and uninfected mice. The cluster analysis was able to classify these compounds into three clusters: I) octanal, *cis*-verbenol, 2-nonenal, naphthalene, (-)-myrtenol, verbenone, carvone, (E)-2-decenal and tetradecane; II) nonanoic acid, undecanal, geranylacetone and hexadecane; and III) the 18 remaining compounds (Fig 6).

According to the volatile compounds released, uninfected animals Un2/Un3 and infected animals I2/I3 were grouped into clusters I and II, respectively. Uninfected animal Un1 and infected animal I1 were grouped with cluster III (Fig 6).

This result showed that individual variations in volatile compounds profiles occur even in isogenic animals of the same age. Only animals Un1 and I1 presented a higher abundance of volatile compounds and a higher attractiveness than the control in the Y tube olfactometer test (Fig 4).

## Discussion

Host manipulation by parasites, which can enhance transmission, has been an interesting topic of debate. The main point of this debate is whether such strategies are true adaptive manipulations or side effects of infection [36]. A good example of a mechanism by which parasites affect feeding behaviour is the blocked fly hypothesis for the interaction between *Leishmania* and sand flies. When sand flies are infected by *Leishmania* parasites, promastigote secretory proteophosphoglycan gel causes lower blood ingestion from a single host, forcing the insect to feed on multiple hosts and thus favouring the transmission of *Leishmania* [21,22].

Parasites can also change the volatile compounds produced by infected hosts, increasing or reducing the concentration of certain compounds to influence the attractiveness of the host and favour transmission [18,37,38]. Currently, an increasing number of studies have investigated the attractiveness of parasitized hosts to their vectors. For *Plasmodium*, some studies have demonstrated that infected animals are more attractive than uninfected animals to Culicidae [15–18].

With respect to sand flies, it was shown that *L*. *infantum*-infected hamsters presented higher attractiveness than uninfected animals to *Lu*. *longipalpis* [19,20]. In a precursory cross-sectional study, two infected hamsters showed higher attractiveness than uninfected hamsters [19]. Moreover, a more extensive and longitudinal study compared 13 hamsters before and after *L*. *infantum* infection and showed a consistent increase in the attractiveness of six individuals after infection. These results also demonstrate that genetic variability among animals is a relevant factor in attractiveness since in some animals, the attractiveness rate was not changed [20]. Conversely, in the present study, differences in the attractiveness of *L*. *braziliensis*-infected mice to *Ny*. *neivai* were not observed, neither did the blood volume ingested by insects that fed on infected or uninfected mice. Despite individual differences that may exist in the volatile compounds released (as the results of PCA of volatile compounds have shown), we tried to minimize possible distortions by using isogenic animals maintained under the same food conditions. The present study used animals of the same age, which is also a relevant factor for the profile of released volatile compounds [39]. Taking into account the clustering of the volatile compounds from the six animals and the results of Nevatte et al. [20], our use of a cross-sectional design rather than a longitudinal design may be one limitation of this study. In addition, the study of O’Shea et al. [19], which used a cross-sectional design with two infected hamsters, reported differences in attractiveness to *Lu*. *longipalpis*.

The animal model used in the above mentioned studies was the hamster. In the present study, we used BALB/c mice for three main reasons: a) mice are a reliable animal model for *L*. *braziliensis* infection [40]; b) mice have been found to be naturally infected with *L*. *braziliensis* in the wild [41]; and c) mice are normally used to feed *Ny*. *neivai* females in our colony. The blood feeding rate during the attractiveness tests in cages with infected and uninfected mice can be considered low, with a mean of 22.5% of successfully blood-fed females. However, this mean is close to that observed during our laboratory routine for maintaining our *Ny*. *neivai* colony, which is approximately 26% (unpublished data).

One possible hypothesis for the discrepancy between our results and data from the literature concerning *L*. *infantum* and *Lu*. *longipalpis* [19,20] is that the discrepancy is due to differences in vector-parasite-host interactions among the studies, i.e., different species of *Leishmania*, sand flies, and hosts. It is well known that there are ecological and epidemiological differences between visceral and cutaneous leishmaniasis, especially concerning the reservoirs of the parasites [42]. With respect to the transmission of visceral leishmaniasis, the findings obtained concerning the attractiveness of animals infected with *L*. *infantum* [20] are in accordance with the ecological pattern of aggregated distribution, in which 20% of hosts are responsible for at least 80% of transmission [43]. In addition, some studies have demonstrated the relevance of dogs as the main reservoirs [44], but for cutaneous leishmaniasis, there is still a gap in our knowledge of the important reservoirs. It has been strongly suggested that *L*. *braziliensis* is a multi-host parasite [45,46] and does not have a specific and main reservoir. The system of multi-host parasites is complex because many factors may influence transmission. Therefore, it is premature to infer the impact of our results on the transmissibility pattern of cutaneous leishmaniasis caused by *L*. *braziliensis*. It is possible that a larger number of studies in chemical ecology may help to elucidate the epidemiological complexity of cutaneous leishmaniasis.

The mechanism by which parasites change host odours is well studied in some *Plasmodium* species. It is already known that hosts infected with *Plasmodium* spp., such as mice and humans, release different patterns of volatile compounds [18,47]. Physiologically, it is known that red blood cells infected with *P*. *falciparum* produce a precursor that is responsible for increased production of CO_2_, aldehydes, and monoterpenes, increasing both attractiveness and susceptibility of infection to *Anopheles gambiae* [48]. These terpenes have already been identified in cultures of cells infected with *P*. *falciparum* and elicit positive electrophysiological responses in *An*. *gambiae* [49]. Another possible mechanism by which *Plasmodium* spp. alter host odour is by modifying the skin microbial profile [50].

In leishmaniasis, different volatile profiles are exhibited by *L*. *infantum*-infected hosts in comparison to those of uninfected animals [19,23]. Of the 31 compounds identified in our study, some have already been found in samples from mice [51], dogs [23] and humans [35,52–54]. PCA of these compounds did not demonstrate a complete differentiation or separation of the volatile compounds patterns of infected and uninfected mice, revealing a strong influence of individual variability among the animals used, despite the use of isogenic mice. This variability could explain the lack of differences in attractiveness to sand flies.

Interestingly, although there were no differences in the volatile compounds profiles of infected and uninfected mice, 11 compounds presented a higher relative area in the uninfected mice: octanal, limonene, nonanal, *cis*-verbenol, 2-nonenal, naphthalene, (-)-myrtenol, verbenone, carvone, (E)-2-decenal, and tetradecane. Among these 11 compounds, PCA and cluster analyses showed a positive correlation of nine compounds (octanal, *cis*-verbenol, 2-nonenal, naphthalene, (-)-myrtenol, verbenone, carvone, (E)-2-decenal and tetradecane) with the group of uninfected mice. The presence of some compounds in higher abundance in uninfected hosts has already been reported for humans in comparison to individuals infected with *P*. *falciparum* [35].

Among the nine compounds in cluster I that were more abundant in uninfected mice, *cis*-verbenol and verbenone presented repellent activity against *An*. *gambiae* [55,56]. In addition, octanal presents a weak repellent activity against *Culex quinquefasciatus* and a dose-dependent repellent effect on *An*. *gambiae* and *Ae*. *aegypti* [57].

In relation to the other compounds that were more abundant in the infected animals, four compounds (nonanoic acid, undecanal, geranylacetone and hexadecane) were shown to be associated with this group of animals (I2/I3; cluster II). Geranylacetone is a compound that is found in human skin emanations and presents repellent activity against *Ae*. *aegypti*, *An*. *gambiae* and *Cu*. *quinquefasciatus* [57,58].

Although the infected and uninfected mice in cluster III did not demonstrate differences in volatile compounds profiles, the abundance of 18 compounds was higher than in clusters I and II, which could be a possible explanation for the higher attraction of sand flies to these two groups of animals (I1 and Un1) than to the control, compared with the other infected and uninfected mice used in Y-tube olfactometer trials.

Some of the identified compounds influence attractiveness to different insect species of health importance. Compounds such as octanal, nonanal, decanal, undecanal, and dodecanal induce an attractiveness response in haematophagous insects, such as *Triatoma infestans*, *Cu*. *quinquefasciatus*, and *An*. *gambiae* [59–62]. For sand flies, nonanol induces an activation response but not an attraction response for both *Lu*. *longipalpis* and *Ny*. *neivai* in wind tunnel tests [14,63].

The presence of compounds with repellent and/or attractiveness activity in both groups of animals (infected and uninfected), together with the individual variations cited above, could also explain the absence of a difference in attractiveness between infected and uninfected mice. Further tests should be performed after, changing the animal model or even the *L*. *braziliensis* strain to evaluate the attractiveness of infected hosts to *Ny*. *neivai* and to identify volatile compounds released by these hosts.

## Conclusions

The number of studies investigating the attractiveness of *Leishmania-*infected hosts to sand flies is still scarce, with most studies focused on visceral leishmaniasis (VL) and *Lu*. *longipalpis*. This is the first study to investigate the attractiveness of infected hosts in the context of ACL. Contrary to previous studies of VL, our results showed no differences in the volatile compounds released by infected hosts or in the attractiveness of these hosts when examined by behavioural experiments. The ecological and epidemiological contexts of VL and ACL are distinct; thus, these peculiarities may explain the present results. However, further longitudinal studies are necessary to evaluate intrinsic differences among infected animals related to attractiveness to sand flies.

## Supporting information

S1 Table Raw data of attractiveness tests in Y-tube olfactometer for uninfected and infected mice. (DOCX)

S2 Table Volatile compounds tentatively identified based on retention time (in ascending order) and their respective experimental and literature retention indexes. (DOCX)

S1 Fig Ordination of volatile compounds tentatively identified after Ward’s cluster analysis. (TIF)
