# Supplementary material for: Influence of Leishmania (Viannia) braziliensis infection on the attractiveness of BALB/c mice to Nyssomyia neivai (Diptera: Psychodidae)
Source: PLoS One. 2019 Apr 1;14(4):e0214574. doi: 10.1371/journal.pone.0214574 (PMC6443145; doi:10.1371/journal.pone.0214574)
Supplement: S1 Table — (DOCX) [file pone.0214574.s001.docx]

Table S1.

| Mice | Activated | Attracted mice | Control |
| --- | --- | --- | --- |
| I1 | 23 | 16 | 4 |
| Un1 | 19 | 13 | 4 |
| I2 | 20 | 10 | 7 |
| Un2 | 18 | 7 | 8 |
| I3 | 21 | 8 | 11 |
| Un3 | 20 | 7 | 8 |
| Total Infected | 64 | 34 | 22 |
| Total Uninfected | 57 | 27 | 20 |
