# Supplementary material for: Influence of Leishmania (Viannia) braziliensis infection on the attractiveness of BALB/c mice to Nyssomyia neivai (Diptera: Psychodidae)
Source: PLoS One. 2019 Apr 1;14(4):e0214574. doi: 10.1371/journal.pone.0214574 (PMC6443145; doi:10.1371/journal.pone.0214574)
Supplement: S2 Table — (DOCX) [file pone.0214574.s002.docx]

**S2 Table**

| Peak no. | Retention time (min) | Compound | RI exp | RI lit |
| --- | --- | --- | --- | --- |
| 1 | 7.67 | octanal | 1,008 | 1,002 |
| 2 | 8.69 | limonene | 1,036 | 1,034 |
| 3 | 11.95 | nonanal | 1,107 | 1,104 |
| 4 | 14.05 | *cis*-verbenol | 1,150 | 1,145 |
| 5 | 14.77 | 2-nonenal | 1,163 | 1,163 |
| 6 | 15.43 | 1-nonanol | 1,175 | 1,172 |
| 7 | 16.06 | naphthalene | 1,186 | 1,181 |
| 8 | 16.95 | (-)-myrtenol | 1,200 | 1,199 |
| 9 | 17.36 | decanal | 1,208 | 1,208 |
| 10 | 17.61 | verbenone | 1,213 | 1,212 |
| 11 | 19.59 | carvone | 1,248 | 1,249 |
| 12 | 20.61 | (E)-2-decenal | 1,265 | 1,266 |
| 13 | 21.29 | nonanoic acid | 1,276 | 1,278 |
| 14 | 22.84 | tridecane | 1,299 | 1,300 |
| 15 | 23.34 | undecanal | 1,308 | 1,306 |
| 16 | 26.72 | *cis*-8-undecen-1-al | 1,364 | 1,365 |
| 17 | 29.01 | tetradecane | 1,399 | 1,400 |
| 18 | 29.71 | dodecanal | 1,408 | 1,408 |
| 19 | 33.26 | geranylacetone | 1,450 | 1,455 |
| 20 | 35.29 | dodecanol | 1,471 | 1,473 |
| 21 | 37.96 | pentadecane | 1,498 | 1,500 |
| 22 | 51.00 | hexadecane | 1,599 | 1,600 |
| 23 | 52.07 | tetradecanal | 1,615 | 1,612 |
| 24 | 57.50 | heptadecane | 1,699 | 1,700 |
| 25 | 58.20 | pentadecanal | 1,715 | 1,715 |
| 26 | 58.80 | 6-phenyldodecane | 1,730 | 1,725 |
| 27 | 61.67 | octadecane | 1,798 | 1,800 |
| 28 | 62.28 | hexadecanal | 1,817 | 1,821 |
| 29 | 64.38 | hexadecanol | 1,880 | 1,880 |
| 30 | 64.96 | nonadecane | 1,898 | 1,900 |
| 31 | 67.78 | eicosane | 1,996 | 2,000 |
