# Supplementary figures and images for: Influence of Leishmania (Viannia) braziliensis infection on the attractiveness of BALB/c mice to Nyssomyia neivai (Diptera: Psychodidae)
Source: PLoS One. 2019 Apr 1;14(4):e0214574. doi: 10.1371/journal.pone.0214574 (PMC6443145; doi:10.1371/journal.pone.0214574)

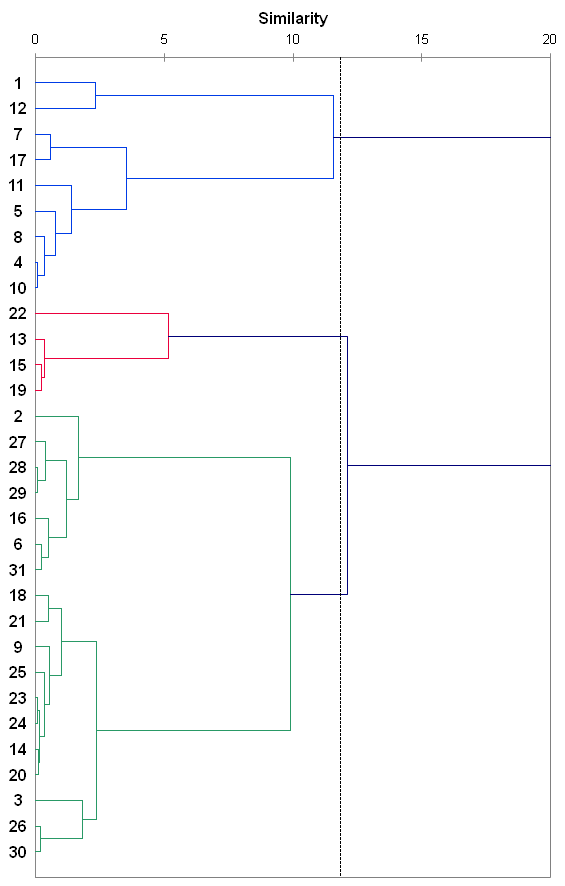

Supplement: S1 Fig — (TIF) [file pone.0214574.s003.tif]
